# Nutritional Practices and Knowledge of Patients with Phenylketonuria

**DOI:** 10.3390/nu17213351

**Published:** 2025-10-24

**Authors:** Eirini Kaloteraki, Eleni C. Pardali, Dimitrios Poulimeneas, Varvara Mylona, Eleni Fotiadou, Kyriaki Papa, Aristea Gioxari, Martha Spilioti, Dimitrios P. Bogdanos, Maria G. Grammatikopoulou

**Affiliations:** 1 Immunonutrition Unit, Department of Rheumatology and Clinical Immunology, Faculty of Medicine, School of Health Sciences, University of Thessaly, Biopolis, GR-41223 Larissa, Greeceelpardali@uth.gr (E.C.P.); 2 Department of Nutritional Science and Dietetics, School of Health Sciences, University of the Peloponnese, Antikalamos, GR-24100 Kalamata, Greece; dpoul@hua.gr (D.P.);; 3 Department of Nutrition and Dietetics, Harokopio University, 70 El. Venizelou Avenue, Kallithea, GR-17671 Athens, Greece; 4 Department of Nutrition and Dietetics, “Attikon” University Hospital, Rimini, GR-12462 Athens, Greece; 5 Nutrition Department, AHEPA University Hospital, Medical School, Aristotle University of Thessaloniki, University Campus, 1 St. Kyriakidi Str., GR-54636 Thessaloniki, Greece; 6 Endocrine Unit, Third Pediatric Department, Hippokration General Hospital, Aristotle University of Thessaloniki, 49 Konstantinoupoleos Str., GR-54124 Thessaloniki, Greece; 7 1st Department of Neurology, AHEPA University Hospital, Aristotle University of Thessaloniki, University Campus, 1 St. Kyriakidi Str., GR-54636 Thessaloniki, Greece

**Keywords:** aminoacidopathy, medical nutrition therapy, metabolic disease, hyperphenylalaninemia, diet therapy, phenylalanine, metabolic control, tyrosine

## Abstract

**Background/Objectives**: Phenylketonuria (PKU) is an inborn error of metabolism (IEM) that requires a specialized medical nutrition therapy (MNT) to maintain blood phenylalanine concentrations within a safe range. This study aimed to assess nutrition practices, knowledge, and PKU diet adherence in patients with PKU. **Methods**: This cross-sectional study included 27 patients (*n* = 19 women) with PKU, recruited from clinics of IEM in Greece, ranging in age between 14 and 60 years, with PKU diagnosis via neonatal screening. Each participant completed the questionnaire independently. For the two patients with age below 18 years old, caregivers provided written informed consent. All participants were questioned regarding their dietary practices, nutritional knowledge, and perceptions. **Results**: More than half (66.7%) of patients complied with the PKU diet and the recommended daily protein substitutes. However, 25.9% reported being unaware of their blood phenylalanine levels, and 40.7% didn’t know how many PKU exchanges they consumed daily. Most patients (88.8%) perceived the recommended PKU diet as “healthy”, and reported feeling well when adhering to it. Several concerns were raised regarding protein substitutes, with 10.5% of patients feeling that the amount of prescribed protein substitutes was too high, while 25.9% perceived it as being too low. Additionally, 14.8% of patients expressed concerns regarding the protein amount required for building muscle mass. Overall, the majority of participants perceived the PKU diet as being adequate in energy, carbohydrates, lipids, and protein. **Conclusions**: Although patients with PKU generally possess a good understanding of PKU nutritional principles, significant potential for improvement in dietary education is apparent. To support optimal management of blood phenylalanine concentrations, it is essential to implement novel communication strategies that facilitate patient adherence to the MNT for PKU. Such strategies should also empower caregivers to provide effective support, including the proper use of protein substitutes and accurate protein exchanges.

## 1. Introduction

Phenylketonuria (PKU), an inborn error of metabolism (IEM), is the most common autosomal recessive disorder of Mendelian phenotype amino acid metabolism. Patients with PKU are unable to efficiently metabolize phenylalanine (Phe), due to phenylalanine hydroxylase (PAH) deficiency [1,2]. In the majority of cases, PKU is the result of missense mutations in the gene encoding PAH, which catalyzes the hydroxylation of Phe to tyrosine (Tyr) [3]. PKU consists of a rare condition, with 0.45 million individuals being affected globally and a prevalence of 1 in every 23,930 live births [2].

Furthermore, PKU belongs to the “toxic accumulation-IEMs”, with the circulating toxin being Phe [3]. The accumulation of Phe can lead to brain dysfunction, growth retardation, epilepsy, intellectual disability, or behavioral issues [1,4]. As a result, Phe dietary restriction consists of the mainstay of treatment for more than 60 years, although pharmacological therapies are also available [1], including Tetrahydrobiopterin (BH4) supplementation, pegylated phenylalanine ammonia lyase (PAL), or the use of large neutral amino acids (LNAAs). Specifically, pegylated PAL is an enzyme that reduces Phe levels in an efficient way, by converting Phe to ammonia and trans-cinnamic acid, independent of PAH activity [5]. It is administered via subcutaneous injection and is indicated for adults with PKU who do not achieve adequate metabolic control through dietary management or sapropterin therapy [5].

The treat-to-target approach aims to reduce and maintain blood Phe concentrations within the treatment range of 2–6 mg/dL (120–360 μmol/L), in order to avoid neuropathogenic complications [6]. Meanwhile, dietary intake must also offer the appropriate amounts of energy, macro- and micronutrients for growth, development, and health attainment. Protein substitutes, which contain vitamins, mineral salts, and n-3 fatty acids, are an integral component of the Medical Nutrition Therapy (MNT) for PKU, based on the patient’s metabolic phenotype and daily Phe tolerance [7,8]. These substitutes consist of low- or Phe-free formulations, like glycomacropeptide (cGMP), and provide all amino acids except for Phe, with the aim of maintaining adequate protein and nitrogen intake to support normal growth and metabolic balance [8]. However, little evidence exists regarding the long-term health benefits of protein substitutes [8]. The difficult aspect is that Phe treatment must be maintained for life, with adherence being assessed frequently [6,9].

Given the rare occurrence of the condition, only a few, underpowered studies have been performed, which indicate that most patients fail to adhere to the treatment [9,10,11,12,13], and thus develop a variety of health issues related to elevated Phe concentrations. Research indicates that patients lack sufficient knowledge of the required targeted blood Phe levels, and they are also unaware of the consequences elevated Phe concentrations have on overall health status [14]. Previous data indicate that patients tend to consume diets low in energy, protein, and fiber, while the intake of carbohydrate is high, posing challenges to optimal metabolic control [15]. Furthermore, a significant number of patients miss their annual appointments, further complicating the dietetic management, while limiting the input needed to develop the knowledge and skills required for effective dietary management [14]. On the other hand, adult patients often report feelings of helplessness and shame regarding their restrictive diet [16]. Overall, dietary adherence appears to be challenging, particularly during adulthood [17], with patients relying mainly on protein supplements to meet their needs for protein and micronutrients [17].

Due to the lack of available literature and the importance of diet therapy in PKU, the present cross-sectional study aimed to assess nutrition practices and perceptions regarding MNT, among patients with PKU.

## 2. Materials and Methods

### 2.1. Sample Recruitment

Consecutive patients with a PKU diagnosis via neonatal screening were recruited from clinics of IEM in Greece, and through Greek and Cypriot patient forums, between December 2023 and May 2024. Of the 28 individuals invited to participate, 27 consented to take part, while one was unable to do so due to neurological complications. The study questionnaire was distributed either electronically via email, or in printed form during outpatient appointments. All participants completed the questionnaire independently. Caregivers provided written informed consent for the two participants below the age of 18, in accordance with ethical research guidelines. Characteristics of the sample are presented in Table 1. Participant age ranged from 14 to 60 years old. No pregnant individuals were included in the sample.

Ethical permissions for the study were granted by the Ethics Committee of the Faculty of Medicine situated at the University of Thessaly (3rd/11 December 2023) and the scientific committee of the AHEPA General Hospital (6th/22 February 2024) in Thessaloniki, Greece. Patients provided informed consent prior to participation.

### 2.2. Questionnaires

For the purpose of the study, questions were selected from one previously published questionnaire used on patients with PKU, with permission [10]. Open-ended questions were omitted from the final questionnaire, which consisted of 34 items, 9 related to diet practices and the rest regarding nutritional knowledge and patients’ perception.

### 2.3. Statistical Analyses

Due to the small sample size, and the rarity of the condition, performing statistical analyses was not deemed as feasible. Small samples limit the ability to detect small or subtle differences and make results less reliable, potentially increasing the risk of false negatives. For this, only descriptive statistics were performed, with categorical variables presented as percentages.

## 3. Results

### 3.1. Dietary Behaviors

Table 2 outlines the nutritional practices reported by patients with PKU. Of the 27 participants included in the study, the majority reported always following the prescribed diet (66.7%), with only 3.7% revealing that they were completely off the prescribed Phe-free diet.

When knowledge of PKU exchanges was assessed, a significant proportion of patients stated lack of knowledge regarding the daily number of recommended (37%) and consumed (40.7%) PKU exchanges.

With regard to protein substitutes, most patients (66.7%) adhered to the prescribed daily amount. However, 7.4% of the participants claimed to be unaware of the number of protein substitutes recommended and/or consumed on a daily basis. A small proportion of participants (11.1%) also reported skipping/forgetting the consumption of protein substitutes every day.

The frequency of protein substitute intake reported by participants was compared to the prescribed protein substitute by their medical doctors. Μost participants followed their prescribed regimen; however, small discrepancies were observed across intake categories. Specifically, the proportion of participants taking their protein substitute once daily and BID was lower than prescribed (−3.7% and −7.4%, respectively), while intake of protein substitutes thrice daily was reported slightly more often than prescribed (+11.1%). The proportion of patients reporting intake of protein substitutes more frequently than four times per day was somewhat higher (+3.7%), compared to prescribed. Finally, the number of patients not knowing how often protein substitutes were consumed on a daily basis was similar or slightly lower (0% and −3.7%, respectively) compared to the answers regarding the prescribed protein substitutes.

With respect to blood Phe concentration targets, none of the participants herein exhibited blood Phe concentrations exceeding 1000 μmol/L, although nearly 1/5 of the sample reported being unaware of their blood Phe levels during the time of the investigation. A total of six patients (22.2%) reported blood Phe concentrations exceeding the recommended target levels, while four patients (14.8%) indicated that they had no defined blood Phe target levels, or were unaware of their blood Phe targets. Notably, these four patients were also unaware of their current circulating Phe levels.

### 3.2. Perceptions Regarding the PKU Diet

**Figure 1:**
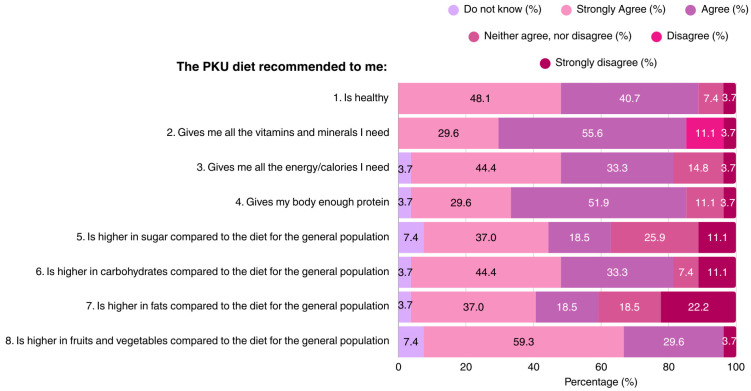
Patient responses regarding their perceptions of the recommended PKU diet (N = 27). PKU: Phenylketonuria.

The majority of patients (88.8%) considered the PKU diet as a healthy diet regime. Most participants believed that the MNT for PKU provides all of the vitamins, minerals, and protein required for health and growth, although opinions differed regarding the carbohydrate, sugar, and fat content of the diet. Approximately 1/3 of the participants regarded the PKU diet as being high in sugar content. The dietary knowledge of patients is outlined in Figure 1.

**Figure 2:**
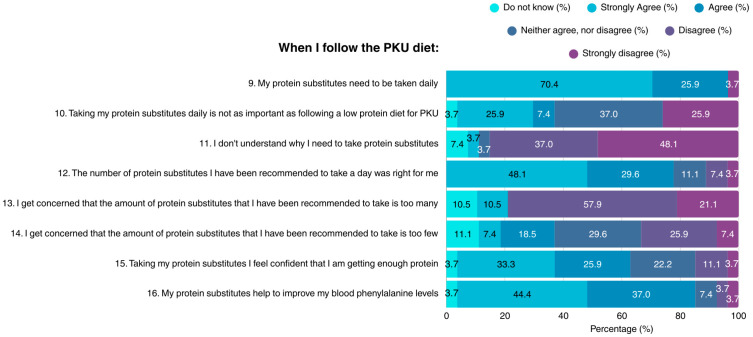
Patient attitudes toward protein substitutes in the PKU diet (N = 27). PKU: Phenylketonuria.

Most participants (96.3%) acknowledged the necessity of consuming protein substitutes on a daily basis when on the PKU diet; however, suboptimal knowledge concerning this topic was demonstrated (Figure 2). The vast majority of patients did not understand why protein substitutes are required, their importance to health, or their role in maintaining blood Phe concentrations within target range. A small proportion of the sample considered the amount of recommended protein substitutes as not suitable for them, while many expressed concerns about either excessive, or insufficient protein intake.

**Figure 3:**
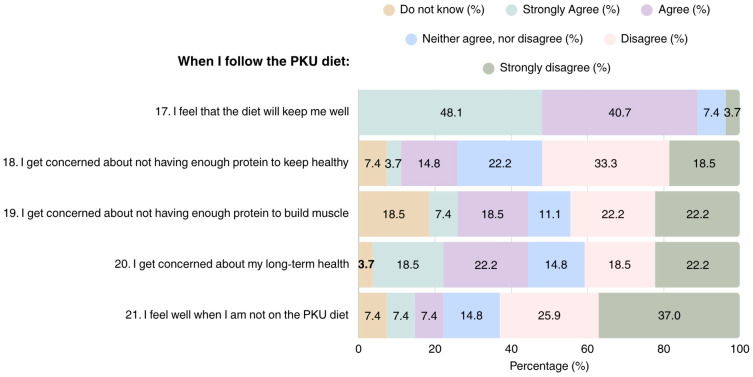
Patient perspectives on overall health in PKU dietary management (N = 27). PKU: Phenylketonuria.

Overall, most patients adhering to the PKU diet considered it as a helpful tool for maintaining health and improving well-being (*n* = 24); however, they also expressed concerns regarding their health in the long term (*n* = 11) (Figure 3). Concerns were also raised regarding the adequacy of the diet in terms of protein content for attaining health (*n* = 5) and muscle development, with 15 patients reporting that they were either unsure, or considered their protein intake was insufficient for muscle development.

**Figure 4:**
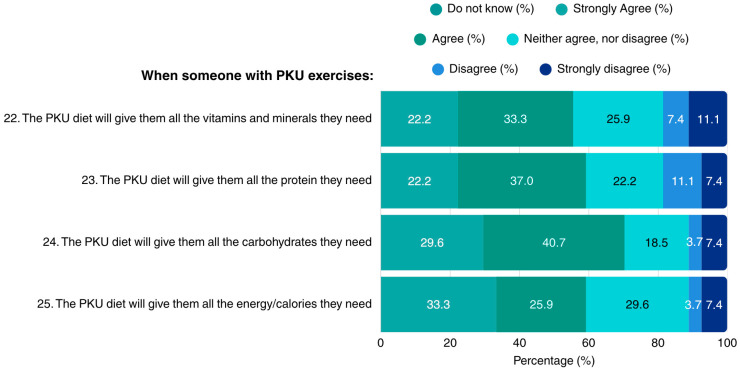
Patients’ opinions on adhering to a PKU diet when exercising (N = 27). PKU: Phenylketonuria.

Regarding the dietary sufficiency of the PKU diet in the context of exercise, patients reported lack of concerns regarding the diet’s adequacy in vitamins, minerals, protein, carbohydrates, and overall energy. Notably, none of the patients indicated lack of knowledge in the questions concerning the MNT for PKU when exercising (Figure 4).

## 4. Discussion

This is the first study conducted in Greece assessing the perception and practices of patients with PKU regarding Phe concentrations and intake, protein substitutes, and adherence to the PKU diet. The results demonstrated that while most patients possessed a good understanding of the PKU diet’s principles and its impact on health, knowledge was not reflected in everyday practice. The majority of participants acknowledged the importance of protein substitutes, although some were either uncertain, or reported lack of knowledge regarding this issue. On the same note, patients also revealed a lack of knowledge regarding the adequacy of protein in the PKU diet for supporting muscle development, while misconceptions emerged regarding the sugar content of the diet.

It is well established that when patients and their caregivers possess adequate knowledge regarding Phe targets, PKU, and PKU MNT, patient adherence to the prescribed treatment increases and PKU outcomes improve [18]. In a study involving children and adolescents, nearly half of the participants were aware of the recommendations regarding daily Phe requirements, while only 17% were aware of the recommended daily protein intake targets, with knowledge increasing with advancing age [16]. The transition from adolescence to adulthood, however, can present additional challenges, including depressive mood, anxiety, and limited autonomy, that may affect patients’ perceptions and behaviors [19]. Interestingly, based on an Italian study, 40% of patients with PKU did not consider PKU to be a disease [20]. This belief was associated with poor dietary adherence and elevated blood Phe concentrations [20]. Moreover, in a study of adult patients, 68% of participants reported not being informed by their medical doctors regarding the risks associated with elevated blood Phe levels [14]. In the present sample, between 11% and 37% of participants either demonstrated limited knowledge, or disagreed with the statement that the PKU diet is healthy and nutritionally adequate. This lack of knowledge might explain why some patients are uncertain about the appropriateness of the PKU diet. Furthermore, PKU exchanges are an integral part of the PKU therapeutic scheme, aiming to keep circulating blood Phe levels as low as possible. Interestingly, more than 1/3 of patients herein reported being unaware of the daily prescribed and consumed number of PKU exchanges. Such misconceptions highlight the importance of providing continuous, tailored education and counseling to both caregivers and patients, to ensure that individuals with PKU attain the knowledge and skills necessary to become confident, well-informed, and autonomous adults.

Although approximately 48.1% of participants maintained blood Phe concentrations within the recommended therapeutic range (<600 μmol/L [21]), 25.9% of the sample exhibited Phe levels between 600 and 1000 μmol/L, indicative of suboptimal metabolic control. Additionally, about one quarter of the sample (25.9%) reported being unaware of their current Phe concentrations. A particularly intriguing finding was that more than 1/5 patients reported higher Phe circulating levels than recommended. Previous reports have shown that adults generally exhibit less effective Phe control compared to younger individuals [22]. In addition, herein, four adult patients were unaware of both their target levels and their current Phe concentrations, reflecting the broader issue of adult patient suboptimal control [23]. Greater circulating blood Phe levels have also been associated with mood swings [24,25] and impaired cognitive function, which in turn, may contribute to dietary and treatment non-adherence, thereby perpetuating a self-reinforcing cycle of poor metabolic control [26].

In the present study, almost all (89%) patients perceived the PKU diet as being useful for health attainment and maintenance, and about 63% reported feeling better when adhering to it. Despite this, one-third of the patients did not follow a PKU diet pattern, raising concerns about the diet’s practicality and easiness to adhere to. According to a recent systematic review [27], four main domains influence dietary adherence in PKU, namely (i) family-related factors, (ii) patient-specific factors, (iii) environmental factors, and (iv) treatment-related factors. Along with traditional barriers [26], the practical challenges associated with the integration of protein substitutes into daily life, such as in educational or occupational settings, have also been documented [28,29]. Taken together, these findings stress that although adherence to the PKU diet improves patients’ health, well-being, and overall quality of life [25], there remains a clear need for a more comprehensive approach and education regarding PKU management.

With respect to protein substitutes, the majority of patients (66.7%) consumed the daily protein substitutes as recommended, while 40.7% of the sample reported never omitting the intake of protein substitutes. However, 29.5% admitted to consuming fewer protein substitutes than recommended, while 33.3% believed that protein substitutes were less important than following the PKU diet. Among subjects who consumed less protein substitutes than advised, the majority expressed uncertainty regarding the adequacy of the prescribed protein intake, and considered it as adequate for building muscle mass. Consistent with this, many participants demonstrated a lack of knowledge regarding protein substitutes. Ensuring the provision of an adequate dose of protein substitutes is essential for promoting normal growth, preventing protein deficiency, supplying a high-Tyr source, and maintaining optimal blood Phe control [30]. Several types of protein substitutes are appropriate for exercising patients with PKU, available in either amino acid or cGMP form [30], all showing good adherence rates [31]. These substitutes generally provide more than 70% of the total protein requirements and serve as important sources of energy, vitamins, and minerals, including vitamin B12, for which individuals with PKU are at greater deficiency risk [8,30]. In parallel, total protein intake should supply the age-related safe and adequate levels of protein intake [32]. Previous research has revealed that the availability of adequate protein for building muscle mass is an issue of concern in PKU [10]. In the present sample, among the seven patients concerned with building muscle mass, five were using protein substitutes as advised, one used less than recommended, and one used more than advised. With appropriate guidance and adequate knowledge, patients should feel reassured that dietary treatment does not prevent them from achieving their physical goals, including muscle mass development.

A total of 77.7% of participants agreed that the PKU diet has a greater carbohydrate content than the diet of the general population, and 55.5% believed it is also higher in sugars content. In PKU, natural protein intake is restricted, with the diet often relying heavily on low-protein sources, which typically contain a greater carbohydrate content [33]. As a result, increases are noted in the glycemic load of the PKU diet, potentially impairing insulin sensitivity and raising blood triglyceride levels [33,34]. Ideally, the PKU diet, together with the prescribed protein substitutes and appropriate medical foods, should not exceed the recommended carbohydrate intake, in order to avoid placing patients at additional risk for metabolic complications [4]. This becomes particularly important, given that nearly half (52.8%) of the patients herein were living with overweight or obesity. Furthermore, attention should be given to the consumption of dietary supplements that may contain aspartame—including those used for weight control—as this may induce a rise in blood Phe concentrations [15].

In PKU, lifelong MNT necessitates multidisciplinary team support, adherence to the Phe-free diet, and frequent follow-ups [35]. In parallel, it is important to establish effective channels of communication and support for patients and their parents/caregivers. Evidence suggests that printed consultation materials and recommendations, such as in a filofax-type folder format, are preferred by patients, and that education tailored to individual needs is more effective, even for improved adherence to protein substitutes [36]. Furthermore, patients express a preference for in-person appointments with their physician and dietician, or a combination of in-person and remote appointments, as they believe that this scheme is more effective for managing their symptoms and facilitating the development of a positive relationship [14].

### Study Strengths and Limitations

The current study has several limitations. As a cross-sectional study, it does not allow for the establishment of causal relationships between knowledge, perceptions, and dietary practices. Additionally, the small sample size restricted the use of statistical analyses that could have provided deeper insights. However, the rarity of the condition does not allow for large sample sizes, with most of the relevant published research having used similar sample sizes [17,35]. Moreover, given the rarity of the condition, a mixed adolescent and adult sample was recruited. In addition, the fact that patients completed the questionnaires independently may have introduced bias. Some participants may not have fully understood the questions, or perceived them wrong. Furthermore, by including only participants capable of independent completion, individuals with neurological or developmental difficulties may have been excluded. Responses may have also been influenced by social desirability, or resulted in inconsistent data, rather than accurately reflecting participants’ true experiences. However, this limitation also underscores the need for future studies to implement structured support, or alternative administration methods to ensure inclusivity and data reliability.

The questionnaire used in the present study did not include open-ended questions and has not been formally validated, which may limit the reliability and generalizability of the findings. However, the instrument was adapted from previously published research. Although no inferential statistical analyses were performed, the descriptive presentation of responses provides valuable insight into participant perceptions and knowledge. These findings offer a preliminary understanding of the topic and may serve as a foundation for future research involving validated instruments, larger sample sizes, and more robust statistical analyses, to confirm and expand upon the current results.

Despite these limitations, this is one of the few studies assessing dietary adherence and patient perceptions regarding the MNT for PKU. Additionally, it consists of the first study conducted in Greece examining the perceptions and practices of patients with PKU regarding Phe targets, MNT for PKU, and protein substitutes. The findings highlight persistent gaps in understanding PKU therapy even among adults, emphasizing the need for tailored, age-appropriate educational strategies. Future studies should consider collecting objective, longitudinal Phe measurements to enable a more comprehensive analysis of metabolic control and its relationship with patient-reported outcomes. Developing innovative methods of nutrition education and support, targeted not only at patients but also at caregivers, could improve dietary adherence and empower patients to make informed decisions that positively impact long-term health and quality of life.

## 5. Conclusions

Although most patients have a solid understanding of the PKU diet and its impact on health, this knowledge does not always translate into routine daily practice. Many patients still struggle to adhere to the PKU dietary principles, are unaware of their blood Phe concentrations, and underestimate the importance of protein substitutes. Beyond the medical challenges, these individuals face numerous social and practical difficulties, and their daily lives could be improved.

While most patients report feeling better when following a PKU diet, a significant gap remains between communication and guidance. Efforts should focus on establishing clear communication channels that allow patients and caregivers to connect effectively with healthcare teams, highlight the essential role of protein substitutes in both daily life and physical activity, and ensure products are clearly labeled as low-protein and low-Phe. By bridging these gaps, we can help patients translate knowledge into action, improve adherence, and enhance overall quality of life.

## Figures and Tables

**Table 1:** Characteristics of the sample (N = 27 patients with PKU) *.

Women/men (*n*, %)	19 (70.4%)/8 (29.6%)
Age (years)	31.85 ± 11.2
Body weight (kg)	76.1 ± 19.4
Stature (cm)	166.3 ± 10.2
BMI (kg/m^2^)	26.2 ± 7.6
Weight status (normoweight/overweight/obesity) (*n*)	13/6/8
Marital status (single/divorced/married/other) (*n*)	16/1/8/2
Higher education attained (secondary/tertiary/post-graduate) (*n*)	9/9/9

BMI: body mass index; PKU: phenylketonuria; SD: Standard deviation. * Data are presented as mean ± SD or as counts (*n*) with their respective percentages (%).

**Table 2:** Nutritional practices of patients with PKU (N = 27).

Question	Answers (% of Positive Answers)
1. PKU diet pattern	(a) Always on a diet (66.7%)
	(b) Return to diet on occasions (29.6%)
	(c) Off diet (3.7%)
2. Number of PKU exchanges recommended each day	(a) ≤5 (7.4%)
	(b) >5–10 (25.9%)
	(c) >10–15 (11.1%)
	(d) >15–20 (14.8%)
	(e) >20 (3.7%)
	(f) Do not know (37%)
3. Number of PKU exchanges consumed each day	(a) ≤5 (7.4%)
	(b) >5–10 (14.8%)
	(c) >10–15 (22.2%)
	(d) >15–20 (7.4%)
	(e) >20 (7.4%)
	(f) Do not know (40.7%)
4. Frequency of protein substitute advised per day	(a) Once/daily (3.7%)
	(b) Twice/daily (11.1%)
	(c) Thrice/daily (66.7%)
	(d) ≥4 times/daily (7.4%)
	(e) Do not know (7.4%)
	(f) Other (3.7%)
5. Frequency of protein substitute taken per day	(a) Once/daily (7.4%)
	(b) Twice/daily (18.5%)
	(c) Thrice/daily (55.6%)
	(d) ≥4 times/daily (3.7%)
	(e) Do not know (7.4%)
	(f) Other (7.4%)
6. Daily protein substitute taken vs. advised	(a) less than recommended (29.6%)
	(b) as recommended (66.7%)
	(c) more than recommended (3.7%)
7. Frequency of missing some/all protein substitute	(a) Every day (11.1%)
	(b) 2–4 times per week (14.8%)
	(c) Once weekly (14.8%)
	(d) Once a fortnight (7.4%)
	(e) Less than once a fortnight (11.1%)
	(f) I never miss taking my protein substitute (40.7%)
8. Blood Phe target level advised	(a) Less than 300 μmol L^−1^ (7.4%)
	(b) Between 120–360 μmol L^−1^ (18.5%)
	(c) Between 120–600 μmol L^−1^ (29.6%)
	(d) Less than 700 μmol L^−1^ (18.5%)
	(e) Less than 1000 μmol L^−1^ (7.4%)
	(f) I do not have a Phe target (11.1%)
	(g) I do not know (7.4%)
9. Last reported blood Phe concentrations	(a) Phe ≤ 600 μmol L^−1^ (14.8%)
	(b) Phe < 120 μmol L^−1^ (7.4%)
	(c) Phe between 120–360 μmol L^−1^ (11.1%)
	(d) Phe between 360–600 μmol L^−1^ (14.8%)
	(e) Phe > 600 μmol L^−1^ (3.7%)
	(f) Phe between 600–1000 μmol L^−1^ (22.2%)
	(g) Phe > 1000 μmol L^−1^ (0.0%)
	(h) I do not know (25.9%)

Phe: phenylalanine; PKU: phenylketonuria.

## Data Availability

The datasets collected for this manuscript are accessible from the corresponding author upon reasonable request.

## Abbreviations

The following abbreviations are used in this manuscript:

cGMP	Glycomacropeptide
IEM	Inborn Error of Metabolism
LNAAs	Large neutral amino acids
MNT	Medical nutrition therapy
Phe	Phenylalanine
PAL	Phenylalanine ammonia lyase
PAH	Phenylalanine hydroxylase
PKU	Phenylketonuria
BH4	Tetrahydrobiopterin
Tyr	Tyrosine
